# Supplementary material for: “What Bothers Me Most Is the Disparity between the Choices that People Have or Don’t Have”: A Qualitative Study on the Health Systems Responsiveness to Implementing the Assisted Decision-Making (Capacity) Act in Ireland
Source: Int J Environ Res Public Health. 2020 May 9;17(9):3294. doi: 10.3390/ijerph17093294 (PMC7246817; doi:10.3390/ijerph17093294)
Supplement: Supplementary file 1 [file ijerph-17-03294-s001.pdf]

International Journal of
Environmental Research and Public Health

MDPI

1. Narrative Interview Guide for Older People including those with a diagnosis Of Dementia

Start the interview with an open-ended question about an experience in a care setting as a patient

1.1. Introduce the scenario:

Let's say you have come into the hospital because you are feeling very unwell and it is discovered that you have an infection. You are given antibiotics to help treat your infection and are admitted into the hospital for a few nights to see how you get on. While the doctors are treating your infection they find out you have anaemia. Anaemia is a condition which effects your blood and might cause you to feel tired or generally unwell. It can have many different causes. Some of these causes can be serious. Some of the causes might not be too serious. Sometimes the doctors can't find a cause.

In order to try to find out what is causing your anaemia the doctors could run some tests. These tests could be a blood test or they may want to use a camera to have a look inside you – the camera might have to go through your mouth. The tests would mean that you would have to stay in hospital for longer.

So, the decision that you and your care team have to make now is: Should you go ahead with these tests to find out what is causing your anaemia? Or should you go home and visit your GP who will keep an eye on you for the next few weeks to see if your condition gets any worse?

1. What information would you need to make a decision? How much information would you need?
2. How would you like to receive information? In writing or a conversation? Or both?
3. Who would be best to give you this information?
4. Would you want to have anyone else be present while deciding? Perhaps, someone to talk to? Who would this person be? How would he/she/they help you?
5. What else would be important to help you make a decision?

Now imagine that you decided to go ahead with the tests and the doctors discovered that the anaemia is not caused by anything too serious. However, if you wanted to treat it, it would require blood transfusions.

1.2. So, the decision that you and your care team have to make now is:

Do you want to go ahead and treat the anaemia through receiving blood transfusion? Or should you refuse the treatment and live with the anaemia which causes you to feel tired and a bit run down?

1. What information would you need to make a decision? How much information would you need?
2. How would you like to receive information? In writing or a conversation? Or both?
3. Who would be best to give you this information?
4. Would you want to have anyone else be present while deciding? Perhaps, someone to talk to? Who would this person be? How would he/she/they help you?
5. What else would be important to help you make a decision?

1.3. Additional enquiries

What about the environment? Would you like to discuss that in a specific setting? What about the noise, lights and spaces?

2. Narrative Interview Guide for Family Carers

2.1. Phase One: Initiation of story

"I'm meeting with family carers of older people, like you, who have recently had an experience of bringing their relative/friend into hospital. I'm doing this so I can better understand what that experience was like for you. I'm particularly interested in how family carers are included in the decision-making or care planning for their family member.

I want to hear your story of a recent experience where you accompanied your relative/friend in hospital and what that experience was like for you as their family carer. Take your time. We've got as much time as you need for this and start wherever you like. I'll listen first, I won't interrupt and I may take a couple of notes that I'll ask you questions about later. Please take the time that you need and as I said I won't interrupt you."

2.2. Phase Two: Main narration

Once the main narration starts, the participant will tell their story without interruption. During this narration the interviewer will engage in active listening as well as encouragement to continue the narration with non-verbal or para-linguistic signals of attentive listening ('hmm', 'yes', 'I see'). The interviewer may take notes during this active listening for later questioning.

Once the participant has come to the end of his/her story the interviewer will probe for anything else:

'Is there anything else you would like to say about that?'

2.3. Phase Three: Questioning Phase

Questions the interviewer has in relation to the story which were noted during the main narration (phase two) are posed using the language of the participant and the references they used in their narration. The purpose of this questioning phase will be to probe for gaps in the story – these can relate to events that were mentioned in the story as well as to the topics of the research.

Examples of questions will include:

- Could you tell me a little bit more about…?
- What happened before/after…?

The topics important to the research which may be addressed here include:

- Communication of information relevant to the patient's care plan (both from and to the family carer)
- Environment in which care planning conversations were occurring
- Who was present (or not present) at care planning/decision-making moments?
- The will and preferences of the patient in relation to the care plan

2.3. Additional questions and topics

- Describe the nature of the decision/s
- Have you been side-lined out by the HCPs?
- How did you represent your loved one?
- Describe the environment and the people involved (physical space, time and so on) were you physically present?
- Did you have enough information and time to decide?
- Were you involved at some stage of the decision process?
- Did the HCPs listen to you? Did you have a say?
- How did you feel?
- Do you want to add something that you wish had happened to make things better/differently?

3. Narrative Interview Guide Health and Social Care Professionals:

Assisted decision-making (ADM) refers to any process in which an individual is supported, through whatever means necessary, to make a decision for themselves.

In light of this understanding of ADM, can you relate a story from your professional experience, where you have assisted the decision-making of an older patient in your care? This story could be a positive or a negative experience.

In preparing to relate this story we would encourage you to think about the following aspects of ADM:

- Who was involved in the decision-making?
- What information was provided, and how?
- Where did the conversations take place?
- What resources were required?
